# Comprehensive chromatographic analysis of Belumosudil and its degradation products: Development, validation, and In silico toxicity assessment

**DOI:** 10.1016/j.heliyon.2024.e38369

**Published:** 2024-09-24

**Authors:** Awadh M. Ali, Mohammed M. Alanazi, Mohamed W. Attwa, Ibrahim A. Darwish, Hany W. Darwish

**Affiliations:** Department of Pharmaceutical Chemistry, College of Pharmacy, King Saud University, P.O. Box 2457, Riyadh, 11451, Saudi Arabia

**Keywords:** Belumosudil, Quality by design, Stability-indicating analytical method, LC-MS/MS, Degradation products, In silico toxicity prediction

## Abstract

Following ICH guidelines, the stability of Belumosudil, a novel protein kinase inhibitor, was tested under different stress conditions (hydrolytic, oxidative, photolytic, and thermal). A selective and efficient separation of Belumosudil and its degradation products was achieved using a Quality by Design approach. In-silico predictions using Zeneth Nexus® software were employed to assess the compound's degradation under various stress scenarios. The methodology developed through experimental design analyzed crucial process parameters connected with chromatographic systems. Reversed-phase high-performance liquid chromatography with a C18 column and a gradient mobile phase of acetonitrile and 25 mM ammonium hydrogen carbonate buffer (pH 5.6) were utilized. For structural characterization and identification of degradation products, UPLC-quadrupole tandem mass spectrometry was employed. Four distinct degradation products were identified under different stress settings. The method was thoroughly validated, assessing accuracy, selectivity, repeatability, system suitability, and linearity range (5.0–120.0 μg/mL). To predict mutagenicity and toxicity, DEREK Nexus® software was used. Two degradation products were predicted to induce skin sensitization, irritation, and hepatotoxicity in humans.

## 1 Introduction

**Fig. 1:**
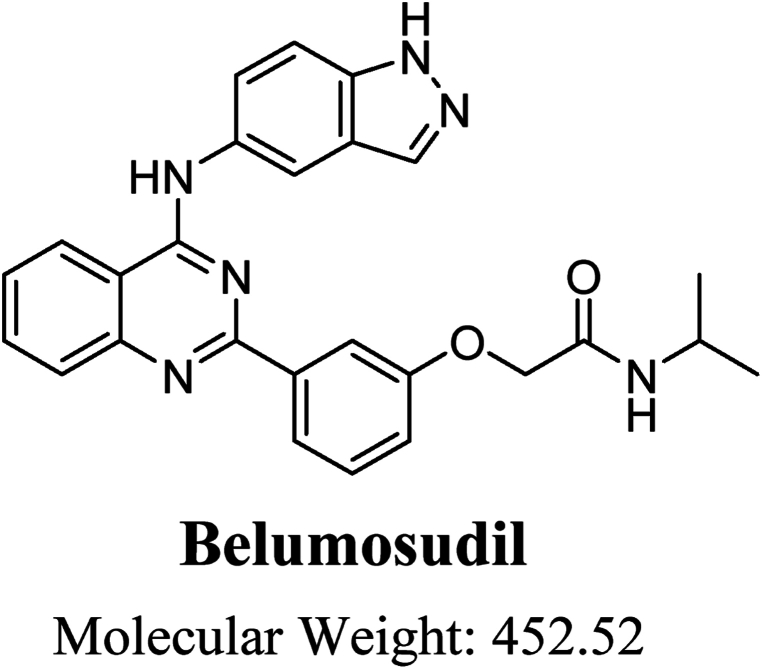
Chemical structure of Belumosudil.

Belumosudil (BEL) is a specific inhibitor of Rho-associated coiled-coil kinase 2 (ROCK2), which has shown great potential as a therapy for inflammatory and autoimmune illnesses, including graft-versus-host disease (GVHD). BEL has the chemical name 2-[3-[4-(1H-indazol-5-ylamino)quinazolin-2-yl]phenoxy]-N-propan-2-ylacetamide. Its structure is shown in Fig. 1. Its first clearance was obtained in the United States in July 2021 for treating adult and pediatric patients aged 12 years and older with chronic GVHD who have not responded to at least two previous courses of systemic therapy. There are limited research articles available regarding the pharmacokinetics, pharmacodynamics, and metabolic profiling analysis [[1], [2], [3], [4], [5]]. Ensuring the stability of BEL during its entire shelf life is essential to preserve its effectiveness, safety, and adherence to regulatory standards [[6], [7], [8]]. It is crucial to establish a strong and dependable approach for monitoring the stability of BEL, utilizing Quality by Design (QbD) principles. This will enable an accurate assessment of its stability and the preservation of its quality characteristics [9,10].

QbD is a methodical strategy that incorporates scientific comprehension, risk evaluation, and quality control across the whole life cycle of product creation [11]. This strategy prioritizes a preventive approach to developing a strong analytical method by thoroughly comprehending the features of the product and the essential aspects that impact its stability. QbD enables the construction of a method that can accurately detect and measure degradation products and contaminants by defining Critical Quality Attributes (CQAs) and Critical Process Parameters (CPPs). This ensures that the method has the capacity to indicate the studied drug stability [6,12].

To develop a stability-indicating method for BEL using the QbD approach, a systematic method is needed. This method should involve a thorough understanding of the physical and chemical properties of the drug substance, formulation parameters, and environmental factors that affect its stability. The effectiveness and accuracy of the procedure in identifying any deterioration or alterations in BEL greatly depends on the systematic identification and optimization of method parameters. The parameters encompass chromatographic settings, including the selection of the column and the composition of the mobile phase, as well as detection methods, such as UV and MS [13,14]. Additionally, stress testing conditions, such as temperature and humidity, are also considered [[15], [16], [17], [18]].

Moreover, the utilization of risk-based approaches within the QbD framework enables the identification and mitigation of potential risks associated with stability assessments. Through the application of Design of Experiments (DoE), it is possible to methodically evaluate and address potential sources of variability and their impact on the method's performance. This methodology (DoE) improves the reliability and uniformity of a stability-indicating method for BEL. This comprehensive method development strategy ensures compliance with regulatory standards and significantly enhances the pharmaceutical development of BEL, facilitating its safe and efficient use in clinical settings [19].

The main goal of stress degradation investigations is to discover and describe possible pathways of degradation, contaminants, and products resulting from degradation [12,17,20,21]. The outcomes derived from stress degradation investigations facilitate the detection of degradation products (DPs) and contaminants, assisting in the determination of degradation pathways and comprehension of the stability characteristics of the drug substance or formulation. Furthermore, these investigations offer essential data required for the creation of reliable and specific stability-indicating methods that can precisely track the stability of drugs during their entire shelf life. Therefore, stress degradation studies are vital for gaining regulatory approval, as they help identify the best storage conditions, shelf life, and packaging to maintain product stability [12,21,22].

Computational methods, including the Zeneth Nexus® software (version 9) and DEREK Nexus® (version 6.2.1), were used to perform in silico experiments to assess the potential degradation pathways and mutagenicity and toxicity of the BEL and its DPs [23]. Zeneth Nexus® software (version 9) is an advanced, knowledge-based in silico program designed specifically for predicting the forced degradation routes of active pharmaceutical ingredients (APIs) under different environmental variables [[24], [25], [26]]. DEREK Nexus® (version 6.2.1), was used to perform in silico experiments to assess the potential mutagenicity and toxicity of the BEL and its DPs. This sophisticated software (DEREK Nexus®) assesses various toxicity endpoints. The outcomes examined encompassed carcinogenicity, chromosome damage, genotoxicity, mutagenicity, hepatotoxicity, irritant effects on the skin, allergic reactions, and ocular toxicity. It is possible that it may be used as a component in a process based on ICH M7 [27]. The results from DEREK Nexus® predict the reverse mutagenicity of bacteria through the Ames test [28], which rely on the careful evaluation of the structural similarity of query molecules and Ame's test outcomes of the predictive model's data points [29]. To the best of our knowledge, there is currently a gap in the literature concerning the development and validation of a published analytical method specifically designed for investigating the stability of BEL and its DPs, as well as their potential toxicity assessment.

## 2 Experimental

### 2.1 Material and reagents

LEAPChem (Hualong, Hanzhou, China) was the supplier of the BEL standard (DD-061215; 98 %). All of the analytical reagent grade substances were obtained from the regional market: sodium hydroxide pellets (NaOH, Merk, Darmstadt, Germany), hydrochloric acid (36 % HCl w/w, Fluka, London, UK), ammonium bicarbonate (NH_4_HCO_3_, Lobachemie, Mumbai, India), acetic acid (99.6 % CH_3_COOH; Winlab, Harborough, UK), Dimethyl sulfoxide (DMSO, 99.9 % (CH_3_)_2_SO; Fisher, Oxford, UK) and hydrogen peroxide (30 % H_2_O_2_ w/w; Avonchem, London, UK). Methanol (MeOH) and acetonitrile (ACN, C_2_H_3_N) suitable for HPLC were purchased from Sigma-Aldrich Company, (West Chester, PA, USA). A Milli-Q Plus filtration system, produced by Millipore (Millipore, Bedford, MA, USA) was utilized to treat and produce the ultra-pure water used in the experiments.

### 2.2 Instrumentation

In order to separate, identify, and quantify BEL and its associated DPs, a high-performance liquid chromatography (HPLC) system from Shimadzu (LC-20AD, Japan) was used. This instrument had a diode array detector (PDA). As a stationary phase, the C_18_ column was used (Zorbax Eclipse Plus C_18_, 250 mm, 4.6 mm, 5 μm; Agilent, Santa Clara, San Francisco, USA). For seeing and processing the output signal, the LCsolution software was used. The samples were dissolved, and the pH of the mobile phase was determined with the use of an ultrasonic bath (Elma S180H; Singen, Baden-Wurttemberg, Germany) and a pH-meter (pH 211; Hanna, Nusfalau, Romania), respectively. Furthermore, to ease the process of heat-mediated hydrolysis, a shaker equipment, namely the Maxi-shake, manufactured by Heto (Allerød, Denmark), was used. The thermally conducted studies involving forced degradation with precise temperature control, were in a Genlab oven from Halton (UK). The weight of both the samples and the standards was determined with the assistance of a Mettler Toledo analytical balance, model B154-S, (Greifensee, Switzerland). photolytic experiments were carried out according to the second option of the ICH guideline Q1B [21], in a photostability chamber model (APT.line® KBF-ICH-720; Binder, Tuttlingen, Germany). The chamber was kept at a temperature of 40 ± 5 °C and a relative humidity (RH)of 30 % ± 3 %. A lighting system that was in accordance with ICH standards was installed in the door of the chamber. This system included a mixture of ultraviolet and white fluorescent bulbs. Additionally, in order to characterize DPs, Electrospray ionization (ESI) was used in the LC-MS/MS investigations that were carried out with the assistance of a Waters UPLC-MS/MS apparatus that was outfitted with an Acquity UPLC (H10UPH) and an Acquity TQD MS (QBB1203). The C_18_ column was used as the stationary phase. After adjusting the MS settings using the IntelliStart® software for BEL, the voltage for the fragmentor was at 40 V, the voltage for the capillary was set to 54 V, the temperature for the capillary was set to 360 °C, and the flow of nitrogen gas from the source was set to 700.0 L/h. During the LC-MS/MS research, these settings are designed to provide the mass detector with the most effective combination of parameters. A nitrogen generator from (Peak Scientific, Inchinnan, UK) was used to produce highly purified nitrogen. A collision gas consisting of argon with a purity of 99.999 % was used in the TQD mass analyzer to affect the fragmentation of analyte ions into smaller pieces. To achieve the required level of vacuum, the TQD mass analyzer was connected to a vacuum pump by Sogevac® (Murrysville, Pennsylvania, US). Under the same experimental circumstances, the spectra were obtained by using the MassLynx 4.1 program (Version 4.1 with the same parameters). In addition, two HPLC columns were investigated. These columns were described as Phenomenex C_18_ (Torrance, California, US), Grace phenyl, and Cyano (Bannockburn, Illinois, United States). The dimensions of these columns were 250 × 4.6 mm, with a particle size of 5 μm.

### 2.3 Software

The Design Expert® processing program (version 12; StatEase Inc., Minneapolis, MN, USA) was used to optimize the mobile phase slope, flow rate, and pH of the buffer system in order to separate a combination of BEL from its DPs.

For prediction of DPs of BEL in silico Zeneth Nexus® software was utilized. The program, created by Lhasa Limited, utilizes information-based transformation to accurately predict the breakdown routes of a given molecule based on its structure. However, Zeneth software may generate a greater number of DPs than what is encountered in reality. The program's functionality relies on incorporating inter-chemical structure, reaction circumstances, maximum number of steps, and probability threshold of chosen query molecules (section 3.6.1).

For prediction of toxicity and mutagenicity of BEL and its DPs another in silico Lhasa limited software, DEREK Nexus®, was used. It was utilized to predict the mutagenicity and toxicity of BEL and its DPs through a knowledge-based technique to make predictions. By evaluating a wide range of endpoints across many species, DEREK Nexus® may help evaluate a chemical's potential dangers, such as its mutagenicity, ocular toxicity, carcinogenicity, skin irritation, hepatotoxicity, and chromosome damage. A setup command will run to commence DEREK's predictions after the chemical structures of BEL and each DP were individually loaded. Section 3.9 provides further explanations of the predictions.

### 2.4 Initial sample preparation and stress degradation investigation

Finding an appropriate solvent to prepare the samples has been a major challenge. When preparing reversed-phase LC samples, ACN and MeOH are often used solvents, although BEL does not dissolve well in them. The solubility of BEL in water is extremely low (<0.1 mg/mL), yet it dissolves in DMSO [30]. In addition, the drug's primary peak is covered by a strong peak in HPLC when DMSO is the sole solvent used. This peak also has a high UV cutoff point. This led to a number of experiments aimed to find the optimal ratio of DMSO to ACN for dissolving BEL. At long last, the 20:80 DMSO: ACN ratio was achieved. In order to minimize the influence of solvent elution in liquid chromatography and the potential for an undesired peak shape, ACN was utilized to adjust the solvent's water content. The solvent was subjected to a 5-min sonication treatment to improve solubility.

To make a 3 mg/mL BEL standard stock solution, 75 mg of the BEL standard was dissolved in 25 mL volumetric flask with a diluent (20:80 % v/v DMSO and ACN, respectively), sonicated for 5 min, and then filled up to the mark with the same solvent. Until it was required, this solution was kept in a freezer at −20 °C.

### 2.5 BEL's forced degradation study

During the stress degradation process, BEL was exposed to acidic hydrolysis, basic hydrolysis, oxidative, thermal, and photolytic conditions in accordance with the ICH standards (Q1A (R2), (Stability Testing of New Drug Substances and Products) and Q1B) (Photostability Testing of New Drug Substances and Products) [12,21]. Every stress test employed a final BEL stock solution concentration of 1.0 mg/mL. In order to evaluate the stability-indicating features of BEL and to profile its DPs, the following forced degradation studies were conducted. The objective was to identify the primary DPs while limiting unwanted reactions and DPs’ deterioration.

#### 2.5.1 Acidic hydrolysis

The sample for acid hydrolysis was prepared by combining 1.0 mL of a stock solution with 1.0 mL of 1.0 N HCl. The sample was subjected to heating at a temperature of 70 °C for a duration of 6 h in a water bath equipped with a shaker. Subsequently, it was allowed to cool down to the ambient temperature and then counteracted with a solution of 1.0 N NaOH to achieve neutralization, resulting in a final concentration of 1.0 mg/mL. A 0.5 mL portion of the previously described solution was mixed with the initial mobile phase in a 5.0 mL volumetric flask to make a final volume of 5.0 mL. The sample underwent filtration and was thereafter introduced into the HPLC system for screening. Subsequently, a blank solution was prepared and subjected to the same treatment as the standard solution.

#### 2.5.2 Basic hydrolysis

A similar procedure was used to prepare the sample for the degradation analysis in basic medium as was done with the acid sample. The process was ended using a solution of 1.0 N HCl. The same steps that were used to produce the standard solution were repeated for the blank solution preparation.

#### 2.5.3 Oxidative stress

The sample's final concentration for oxidative degradation was 1.0 mg/mL. This concentration was obtained by adding 2.0 mL of a 2 % H_2_O_2_ solution to a 1.0 mL stock solution. The drug was left on a benchtop in the dark at room temperature (25 °C) for a duration of 6 h. For comparison, a second sample that did not contain the drug was used and treated in the same way as the standard. Subsequently, the described acid hydrolysis procedure was executed.

#### 2.5.4 Photolytic stress

Two borosilicate glass samples, each containing 10.0 mg of BEL, were weighed. The sample was subjected to (2.4 million Lux-hours) of cool white fluorescent light and (400 W h/m^2^) of UV light, which is double the minimum advised exposure by the ICH guidelines. As a reference, the second sample was kept in the dark and covered with aluminum foil. A photostability chamber model (APT.line® KBF-ICH-720; Binder, Tuttlingen, Germany) coupled with ICH-compliant option-2 illumination in the door lights and a temperature and humidity control system set at 40 ± 5 °C/30 % RH ± 3%RH was used for the investigation. Following the procedures previously mentioned, the samples were treated in the same way.

#### 2.5.5 Ambient and high humidity-related thermal stress

Two samples of BEL, each weighing 10.0 mg, were weighed in borosilicate glass flasks. For seven days, a single sample was heated to 70 °C and desiccated at 70 % RH in an open flask set above a saturated sodium chloride solution. Similar procedures were performed on the second sample, except that humidity was not controlled. Following the procedures detailed in the previous section, the samples were processed in the same way.

### 2.6 Liquid chromatographic conditions

HPLC separation was achieved using a C_18_ column type (Zorbax Eclipse Plus, 250.0 mm, 4.6 mm, 5.0 μm) and two mobile phases, A and B, by the process of gradient elution. The mobile phase A consists of a solution of ammonium bicarbonate buffer with a concentration of 25.0 mM, which has been adjusted to a pH of 5.6 with acetic acid. The mobile phase B consists of ACN. The gradient system was 28–57 % B (0–45 min), 57 to 28 % B (45–50 min) and 28 % B (50–55 min). The temperature of the column was consistently maintained at 25 °C. Both the standard and sample solutions were injected at a volume of 10.0 μL. The absorbance at 337 nm was monitored using a PDA detector.

### 2.7 LC-MS parameters

The molecular mass of both the parent compound and newly formed DPs was determined using the Waters UPLC-MS/MS equipment Acquity UPLC and TQD MS, together with ESI and LC-MS/MS [31]. The cone voltage was adjusted to 40 V, the capillary voltage to 54 V, and the temperature of the probe to 360 °C for the mass detector. The data collection and handling were conducted using Masslynx v.4.1 software. However, two modifications were made. Firstly, the flow rate was reduced to 0.8 mL/min to avoid overwhelming the mass spectrometer. Secondly, the injection volume was decreased to 7.5 μL.

## 3 Results and discussion

The key elements of a QbD oriented analytical approach involve several aspects. These steps include setting up the Analytical Target Profile (ATP), identifying the Critical Quality Attributes (CQAs), using DoE to evaluate Risk Assessment Parameters for screening, optimization, and prioritization, and establishing the parameters to define the Design Space (DS).

### 3.1 Method's ATP

The ATP is intended to be consistent with the objectives of the method, which is to obtain a clear separation between the primary compound (BEL) and its associated DPs with a resolution above 1.5 (baseline separation of peaks). The sensitivity and selectivity of the method are of crucial significance in identifying even trace quantities of these DPs during regular drug analysis. In addition, the PDA detector was used to evaluate the UV spectra of peaks ranging from 200.0 to 400.0 nm in order to standardize the detection wavelength for both the BEL and its DPs.

### 3.2 Method's CQAs

CQAs are essential criteria that are vital for ensuring that the drug's quality is maintained within a predetermined acceptable range or limit [[32], [33], [34]]. These requirements include factors such as separation, identification, accuracy, precision, ruggedness, and robustness. Within the context of chromatographic procedures, distinct CQAs include Resolution (R_s_), column efficiency, capacity factor, peak tailing of analytes, and retention time, among other factors. The chosen CQAs for this research were the resolution between two critical peaks (BEL and process impurity after it U3), capacity factor of the first peak, and retention time of the last eluting peak. The parameters underwent direct modeling utilizing multivariate approaches in the Design Expert® modeling program (version 12). This methodology enabled a thorough examination and representation of these CQAs, so promoting a more profound comprehension of their impact and interconnectedness within the experimental structure.

### 3.3 Risk assessment “screening, prioritization, and optimization”

Risk analysis is a crucial component of the QbD construction. It involves identifying and evaluating variables that affect the performance of a technique and how well it aligns with the ATP. During the process of developing the method, regular risk assessments are carried out to particularly address any variances in laboratory methods and reagent supplies. This preventive strategy enables us to allocate our resources and efforts towards the most crucial domains. Within the scope of HPLC method development, the risk assessment encompasses both qualitative and quantitative factors. Qualitative variables refer to characteristics such as the type of organic phase, column, elution mode (isocratic or gradient), buffer, and detector type. On the other hand, quantitative variables include factors such as the aqueous solution %, modifier %, flow rate, pH, and column temperature.

Using DoE provides a powerful method for thoroughly assessing the combined effects and interactions of these components, allowing for the prediction of their impact on CQAs and the optimization of the chromatographic operation. Therefore, Comprehending the interdependence of these components is vital, it is essential to examine all significant criteria concurrently rather than individually. Nevertheless, implementing a comprehensive factorial design including all factors would provide an excessively vast number of tests, rendering it impractical. Consequently, the risk assessment was conducted in two phases. The first phase consisted of evaluating the key influencing elements that impact CQAs. This was done using the conventional One Factor at a Time (OFAT) technique, with particular emphasis on aspects such as aqueous phase initial percent, gradient time, and column type. Following that, the optimization step included a thorough examination of other factors that affect the process, such as slope of organic phase, flow rate, and pH of buffer system. This examination was conducted using DoE. The systematic method allowed for an in-depth understanding of the significant factors' influence while reducing the need to carry out a large number of trials.

### 3.4 DS “Region of Operable Method”

The term " Region of Operable Method " refers to a multidimensional space where quality assurance operates by integrating and interacting with input variables. The specified design area plays a crucial role in producing a product that meets predetermined quality criteria. Therefore, the design of the analytical method enables the analytical process to enhance its efficiency and reduce its vulnerability to potential risks. When developing a method for HPLC to analyze a pharmacological substance that breaks down under various conditions, it is crucial to establish the specific parameters for controlling the separation process. This might comprise responses such as the resolution between critical peaks and the retention time of the last eluting peak to reduce analysis time, shown either for each specific individual response or collectively across all responses.

The use of DoE in Design Expert® modeling software (version 12) is essential for developing this design environment. It has several functions, such as doing screening experiments, constructing regression models, investigating response surfaces, conducting comparison experiments, and evaluating mixtures. The program utilizes these features to assist in defining and displaying the design possibilities, making it easier to make well-informed decisions throughout the process of developing the method.

### 3.5 Screening and optimization

#### 3.5.1 Screening experiments

**Fig. 2:**
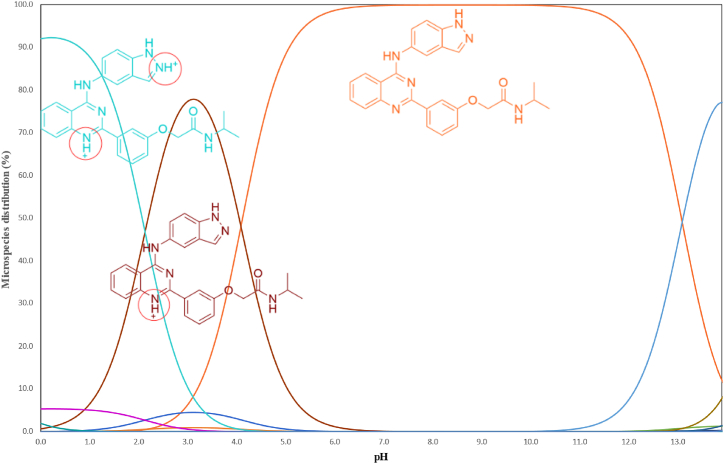
Belumosudil's MarvinSketch-generated pH curves superimposed with the three predominant microspecies. A red circle indicates the parts of the molecule that are ionized. Cyan and dark brown for positively charged, and orange for neutral form.

Initial experiments were carried out using the knowledge obtained from the relevant scientific literature on the BEL. The screening technique included a sequence of independent trials to evaluate distinct components, referred to as OFAT studies. The choice to use reversed-phase LC was based on the molecular structure of BEL and the expected properties of its DPs. Additionally, to avoid any tailing problems caused by the molecule transitioning between several ionized forms at the chosen pH of the mobile phase, it is advisable for the molecule to largely exist in a single ionized state throughout the LC process. Utilizing MarvinSketch software (ChemAxon, Budapest, Hungary), an analysis determined that the molecule BEL displays many ionized forms at different pH levels, as seen in Fig. 2 [35]. Two pH regions were seen: first; from about 5.0 to 11.5, where the majority of BEL would mostly reside in a single ionized state (>90 %). Second at a pH below 0.5, which is impractical.

In order to determine the most appropriate mobile phase, it was decided to examine the aqueous component with a region in the acidic pH. This choice was taken since basic pH values greater than 7.0 will solubilize the stationary phase and are generally unsuitable for most reversed-phase chromatographic columns. Therefore, to achieve better results and presence in more than 98 % of BEL in a single form, pH 5.5 and more was needed. In order to assure compatibility with MS methods used for characterizing DPs, ammonium bicarbonate was chosen as the buffer. At first, MeOH was selected as the organic phase for experimentation. However, longer analysis time was needed and due to the strong elution power and lower back pressure, ACN was chosen. Given the anticipated diverse polarity of BEL and its DPs, gradient elution has been identified as the most efficient method for separating these compounds from one another throughout the chromatographic procedure.

To comprehensively clarify the elution characteristics of BEL from the C_18_ column and to improve the distinction between BEL and its DPs, a set of tests was carried out by using a diverse range of organic phase percentages. The studies included samples that had undergone forced degradation and were combined with the BEL solution. At first, it was seen that the first DP, when compared to BEL, had a greater level of polarity and needed less amount of organic component in the mobile phase in order to be eluted quickly. When the concentration for example of ACN exceeded 35 %, it was eluted together with the solvent front. In contrast, a greater proportion of the organic phase was required in order to achieve effective elution of BEL from the column. Consequently, after several trials, the initial percentage was determined to be 28 % and the final percentage to be not less than 50 % within the time limits for analysis which will maintain the robustness of the method for fluctuation above and below this value. In addition, the objective was to detect small peaks that represent DPs, which can be hidden by abrupt changes in the slope. Therefore, it was considered crucial to have a progressive shift in the slope of the mobile phase. So, it was necessary to further extend the duration of the gradient, which was fine-tuned according to the requirements for liquid chromatography. Nevertheless, there was a specific worry over the threshold pH level at which the solubility of the stationary phase may be negatively affected. Therefore, increasing the pH to 6.5, which was the limit of the stationary phase, and the use of QbD principles enabled the identification of crucial operational parameters for chromatographic separation, guaranteeing an optimum strategy that takes into account the influence of pH fluctuations on the solubility of the stationary phase.

#### 3.5.2 Optimization experiments

**Fig. 3:**
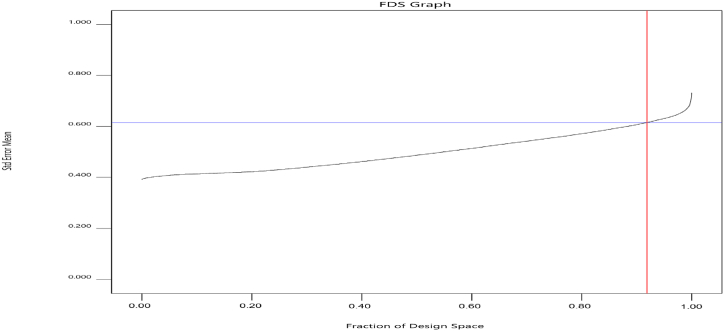
Design space fraction of the developed model. The black line represents the proportion over 92 % (shown by the red vertical line) in relation to the standard error of the mean, which is below 0.615 (represented by the blue horizontal line).

A thorough review of chromatographic settings was carried out utilizing an efficient central composite response surface design in order to discover the most effective circumstances and investigate the combined impact of these factors. This design used three quantitative elements (slope of B%, buffer pH, and flow rate). A total of 25 tests were conducted, changing the parameters at various levels (slope ranging from 55 % to 90 %, pH ranging from 5.5 to 6.5, and flow rate ranging from 0.8 to 1.2. Prior to doing the real testing, the assessment of the model included several essential criteria. Variance inflation factors (VIFs) were computed to measure the extent to which the variance of a model coefficient increased as a result of probable lack of orthogonality related to the design. The VIFs were kept at appropriate levels, ensuring that the model coefficients remained orthogonal to each other and did not have a negative influence on the model fit. Additionally, the design points were limited to less than double the typical values of leverage, ensuring that the model fit was not adversely affected. Furthermore, the absence of any relation among the model coefficients demonstrated the orthogonality of the model. In addition, the fraction of design space (FDS) was used as a visual tool to evaluate the precision and accuracy of the design. The design was deemed robust based on the presence of lower average error scores and consistent error ratings across the factor space. Graphical analysis indicated a reduction in the curvature of the curve, reflecting a flatter form (Fig. 3). This attribute facilitated the assessment of the design space, with a prediction variance less than 0.615 and a 92 % observed proportion inside this particular design. The combination of these factors suggests a well selected and efficient design for the work.

Changing the slope of the organic phase was chosen due to well-established literature that emphasizes their specific impacts on separation. Controlling the pH of the mobile phase is essential for achieving good selectivity of the procedure and enhancing the form of peaks for analytes. Therefore, a constant pH range of 5.5–6.5 was used, using buffers that are compatible with MS. Specifically, a buffering agent of 25.0 mM ammonium bicarbonate was used. Modifying the flow rate has a substantial impact on the separation process. Hence, flow rate modifications were done within the practical limitations of the HPLC equipment, ranging from 0.8 to 1.2 mL/min.

The optimal conditions for achieving the maximum resolution between critical peaks (BEL and process impurity (U3)), retention time of the last eluting peak, and capacity factor of the first peak were established by considering the slope of organic component, buffer pH, and flow rate. This determination included assessing the combination of BEL and its DPs under different stressful conditions employing wide selectivity. The optimization strategy required the implementation of several experiments, including column re-equilibration between each run, blanks, and duplicates. An optimal separation approach should demonstrate enough chromatographic efficiency, successfully isolating BEL from its DPs and accurately differentiating between the different DPs. The UV spectra and peak areas were used to trace the peaks corresponding to BEL and DPs. This analysis included 25 chromatograms from optimization runs. The LCsolution application (version 1.25) was used for accurate chromatogram processing, and subsequently, the results were sent to the Design Expert® modeling software (version 12) for analysis.

The analysis resulted in the choice of the quadratic model as the most appropriate representation for the resolution between BEL and U3 with a resolution of 1.5 or higher (United States Pharmacopeia (USP)). This model was then subjected to a reduction of insignificant model terms (namely AB, BC, B^2^, C^2^). These calculations require keeping any insignificant factor that involved in other interactions as seen here for C-pH which interact with A-Slope (namely AC term). This makes the C term necessary for maintaining the hierarchical structure of the model. The resulting model had statistical significance, as shown in Table 1 by a p-value of less than 0.0001. Additionally, the adopted model showed statistical significance, with a p-value of less than 0.0001, coupled with an adjusted R^2^ value of 0.9478 and a predicted R^2^ value of 0.9287. The ANOVA analysis confirmed the model's statistical significance with a high F-value of 88.14. This F-value suggests an extremely low probability of observing such an F-value due to random variation, at a significance level of 0.01 %. The lack of fit F-value of 2.75 indicates a probability of 6.57 % for a lack of fit F-value of this magnitude to arise from random noise. The presence of a lack of fit is undesirable, as it signifies that the model does not adequately fit the data. This relatively low probability, being less than 10 %, raises concerns regarding the model's fitting capability.

**Table 1:** ANOVA for the reduced quadratic model of the resolution between BEL and Process impurity (the first response).

Source	Sum of Squares	df	Mean Square	F-value	p-value	Notes
Model	7.38	5	1.48	88.14	<0.0001	Significant
A-Slope	5.56	1	5.56	331.74	<0.0001	
B-FR	0.5339	1	0.5339	31.88	<0.0001	
C-pH	0.02	1	0.02	1.19	0.2881	
AC	0.1806	1	0.1806	10.79	0.0039	
A^2^	1.09	1	1.09	65.1	<0.0001	
Residual	0.3182	19	0.0167			
Lack of Fit	0.2265	9	0.0252	2.75	0.0657	Not significant
Pure Error	0.0917	10	0.0092			
Cor Total	7.7	24				

Table 1 displays the significant model terms, such as slope, flow rate, and pH, either independently or interact with each other. Furthermore, certain individual parameters that were squared were shown to be important. The equation, formulated using coded factors, is presented as follows:

Y=3.96−0.5556A+0.1722B+0.0333C−0.1063AC+0.4651A²

**Fig. 4:**
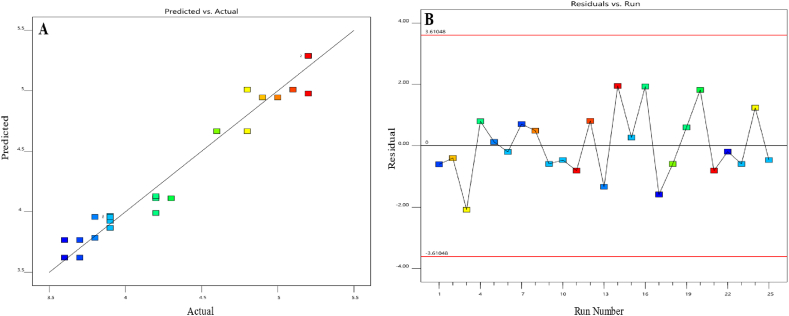
Diagnostic plots for the resolution between Belumosudil and U3. Plot (**A**) displays the predicted response values plotted against the actual response values, while plot (**B**) shows the residuals displayed against the order of the experimental runs. Different colors indicate different runs.

**Fig. 5:**
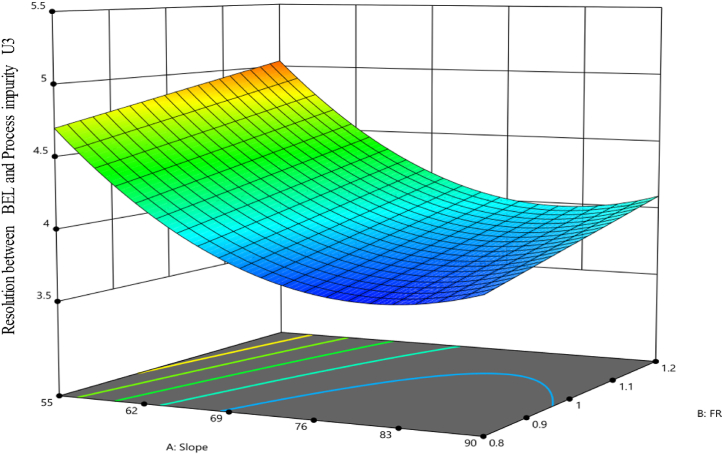
The 3D surface model of resolution between Belumosudil and U3 response at a pH of 5.6.

Fig. 4 (A) shows visual illustrations of the predicted values of the model compared to the actual values, as well as (B) the residual values in relation to the run numbers. When the slope was at a low level and the flow rate was at a high level, while keeping the pH constant at 5.6, a higher resolution between BEL and U3 was seen. The observation is visually shown in Fig. 5 using a 3D surface map. The color gradient used in the graph depicts the numerical values associated with the quantity of resolution attained. The warmer hues, spanning from red to orange, indicate the utmost resolution values for this response. In contrast, the colder blue hues represent the minimum values.

The model with the best data fit on the retention time of the last eluting peak has a quadratic pattern, then the model was subjected to a reduction of insignificant terms. The reduced model is statistically significant with a p-value (<0.0001) and an adjusted R^2^ value of 0.9985. Furthermore, the predicted R^2^ coefficient for this model is 0.9979. The model's F-value of 3176.82 demonstrates its statistical significance, with a very low likelihood of finding such an F-value by chance (only 0.01). The buffer pH was insignificant term in this response. Table 2 presents the main variables of the model, including the main effects and interactions between the slope and flow rate, in addition to squared terms.

**Table 2:** ANOVA for the reduced quadratic model of the retention time for the last eluting peak (second response).

Source	Sum of Squares	df	Mean Square	F-value	p-value	Notes
Model	1817.55	5	363.51	3176.82	<0.0001	significant
A-Slope	1618.8	1	1618.8	14147.25	<0.0001	
B-FR	145.07	1	145.07	1267.79	<0.0001	
AB	4.73	1	4.73	41.34	<0.0001	
A^2^	25.02	1	25.02	218.68	<0.0001	
B^2^	0.7811	1	0.7811	6.83	0.0171	
Residual	2.17	19	0.1144			
Lack of Fit	0.3991	9	0.0443	0.2498	0.9757	not significant
Pure Error	1.77	10	0.1775			
Cor Total	1819.72	24				

**Fig. 6:**
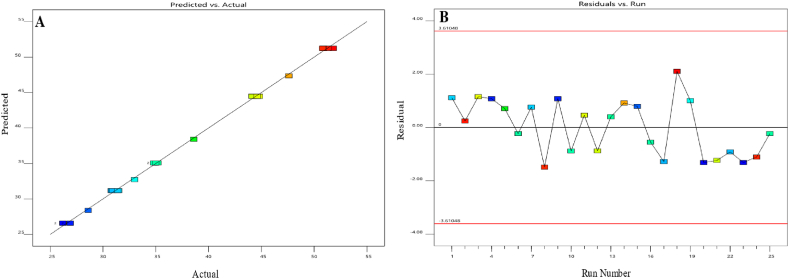
Diagnostic plots of the retention time of last eluting peak response. (**A**) displays the predicted response values plotted against the actual response values; while (**B**) shows the residuals displayed against the order of the experimental runs. Different colors indicate different runs.

**Fig. 7:**
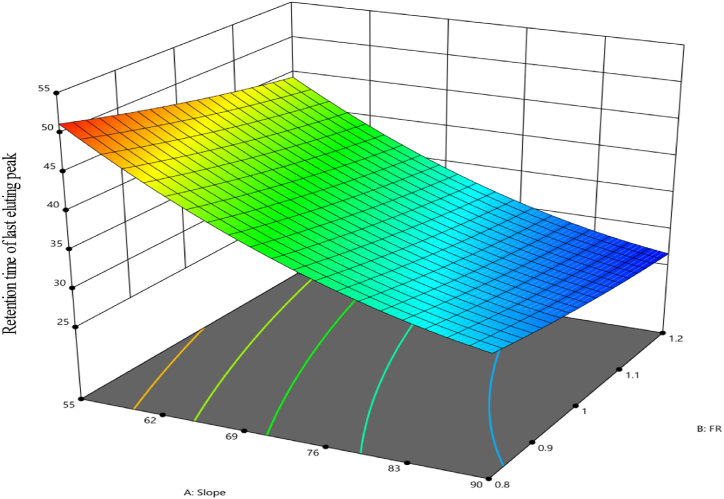
The 3D surface model of retention time of last eluting peak response at a pH of 5.6.

Fig. 6 (A) displays graphical representations that compare the predicted values with the actual values. It (Fig. 6 (B)) also shows the residuals plotted against the experimental run order. Significantly, when the pH is kept constant at 5.6, the use of high slope and higher flow rate values results in a shorter retention time. Fig. 7 graphically demonstrates this trend using a 3D surface plot that employs color coding to depict the numerical values associated with the retention time attained. Colors in the warm spectrum, such as red and orange, represent the longest retention time values. On the other hand, cooler hues of blue indicate lower values. The model is represented by the coded equation shown below.

$$Y=35.07-9.48A-2.84B+0.5437AB+2.79A^{2}+0.4936B^{2}$$

The capacity factor (retention factor) of the first eluting peak initially exhibits a quadratic model, making it the most appropriate model to describe the data. Afterwards, the model was reduced to remove terms that were not significant. The resultant simplified model maintains statistical significance, with a p-value of less than 0.0001 and an adjusted R^2^ value of 0.9808. Furthermore, the predicted R^2^ value for this model is 0.9714. The model's F-value of 175.75 indicates its statistical significance, with a low possibility (0.01) of reaching such a result by chance or due to noise. Notably, the slope, buffer pH and flow rate main effects emerged as significant factors along with their interaction with each other as well as squared terms in this response. Table 3 presents the primary variables within the model.

**Table 3:** ANOVA for reduced quadratic model of the capacity factor for first eluting peak (third response).

Source	Sum of Squares	df	Mean Square	F-value	p-value	Notes
Model	1.18	7	0.1683	175.78	<0.0001	significant
A-Slope	0.0312	1	0.0312	32.64	<0.0001	
B-FR	0.4835	1	0.4835	504.95	<0.0001	
C-pH	0.5168	1	0.5168	539.76	<0.0001	
AB	0.0506	1	0.0506	52.87	<0.0001	
BC	0.0289	1	0.0289	30.18	<0.0001	
B^2^	0.0637	1	0.0637	66.52	<0.0001	
C^2^	0.0112	1	0.0112	11.68	0.0033	
Residual	0.0163	17	0.001			
Lack of Fit	0.0153	7	0.0022	22.63	<0.0001	significant
Pure Error	0.001	10	0.0001			
Cor Total	1.19	24				

The equation, constructed using encoded variables, is stated as follows:

Y=1.12−0.0417A+0.1639B−0.1694C+0.0562AB−0.0425BC−0.1409B²+0.0591C²

**Fig. 8:**
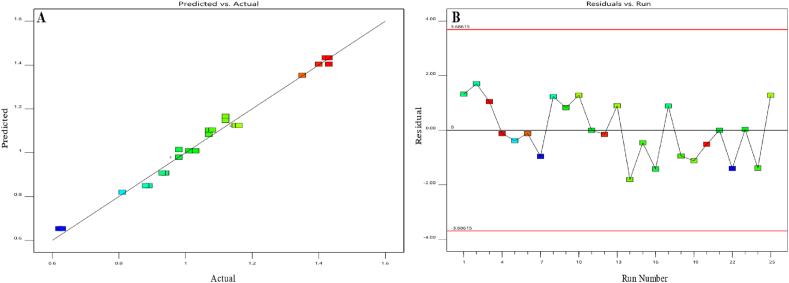
Model diagnostics plots of the capacity factor of the first eluting peak response. (**A**) displays the predicted response values plotted against the actual response values; while (**B**) shows the residuals displayed against the order of the experimental runs. Different colors indicate different runs.

**Fig. 9:**
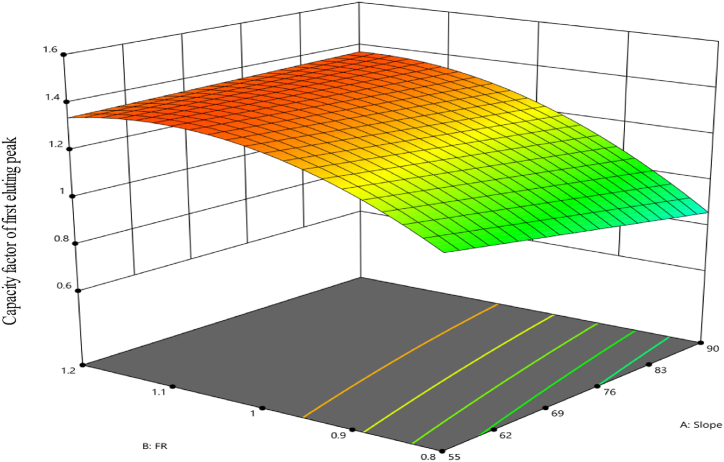
The 3D surface model of the capacity factor of the first eluting peak response at a pH of 5.6.

Fig. 8 (A) presents graphical presentations that compare the predicted values of the model to the actual values. It (Fig. 8 (B)) also shows the residual values in connection to the run sequence. Notably, increasing the slope alone from a lower to a higher value led to a slightly but statistically significant reduction in the response (equation above), while interacting with the flow rate led to a subtle but significant improvement in the capacity factor. Furthermore, the capacity factor was improved by shifting the flow rate from a lower to a higher value. But when the flow rate squared, the response was degraded. Overall, the capacity factor has a nonlinear trend. It first rises from low to high values, then shows a tiny decrease, but constantly stays within acceptable limits at higher flow rate configurations. These alterations were placed while preserving a consistent pH level of 5.6. The data is shown in Fig. 9 using a 3D surface map. The color gradient used in the display represents the numerical values that correlate to the capacity factor attained. The warmer hue color, spanning from red to orange, signifies the largest capacity factor data for this response. In contrast, the colder green hues symbolize the minimum values.

**Fig. 10:**
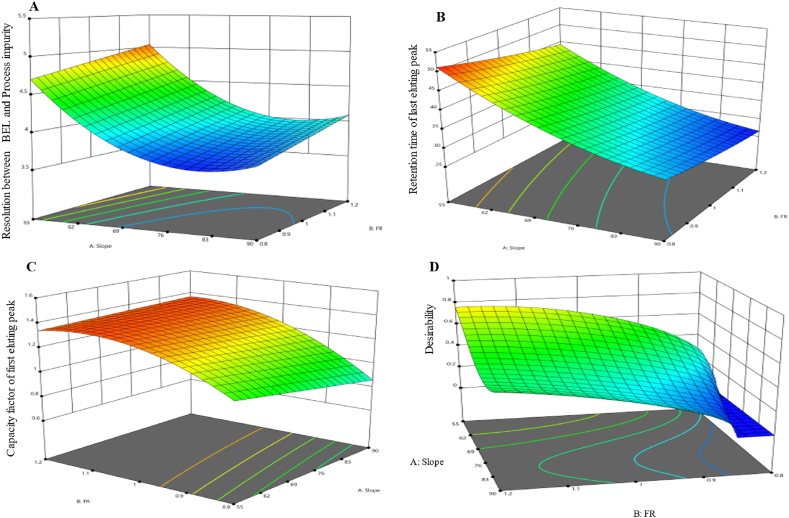
Numerical optimization to (**A**) resolution between Belumosudil and U3, (**B**) retention time of the last eluting peak, (**C**) capacity factor of first peak at flow rate of 1.1 mL/min and pH of 5.6 of the first, second, and third responses, (**D**) The optimal desirability of all responses.

The numerical optimization process aims to maximize the values of both resolutions between BEL and U3 (Fig. 10 (A)) and the capacity factor of the first eluting peak responses (Fig. 10 (C)) but minimize the retention time of the last eluting peak response during assessment (Fig. 10 (B)). At the same time, the goal is to get a desirability score as near to 1 as possible, which indicates the achievement of the most desired result for all responses (Fig. 10 (D)). This optimization method aims to improve overall performance by prioritizing the achievement of maximum values for individual response, while also guaranteeing that the collective desirability approaches an ideal state, Fig. 10. The objective is to achieve an equilibrium where each response is maximized to its fullest capacity, eventually contributing to an overall output that closely fits with the ideal criteria.

#### 3.5.3 Design space

**Fig. 11:**
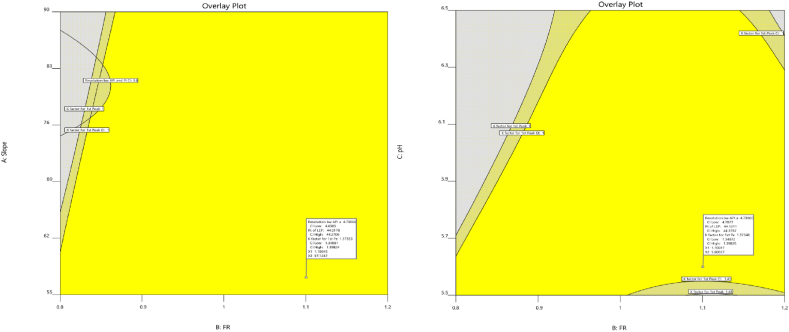
Graphical optimization overlay plot showing the fulfillment of defined conditions. For optimal results, it was essential to increase the resolution between Belumosudil and U3, as well as the capacity factor of the first eluting peak responses, while decreasing the retention time of the last eluting peak response.

The overlay graph offers a concise visual representation that identifies the best area where the requirements for multiple responses may be fulfilled concurrently. Fig. 11 functions as an informative tool that clearly defines the limits of the operable zone in relation to the collective responses. Using a slope of 57 %, a flow rate of 1.1 mL/min, and a pH value of 5.6 places the experimental parameters into the specified area of the created design space. The graphical illustration helps to find and emphasize the exact combination of parameters that are inside the acceptable range, guaranteeing that the selected circumstances satisfy the criteria for all responses simultaneously. It shows all of the scenarios in which the intended responses may be realistically achieved, providing valuable information on the best combinations of parameters to reach the experimental objectives.

### 3.6 Degradation Behavior of the BEL

#### 3.6.1 Zeneth-assisted drug degradation prediction

The degradation prediction analysis for each set of conditions was conducted using the Zeneth program (version 9). The prediction study parameters were conducted for each matrix, using a minimum pathway score of 400 and maximum displayed steps of 2. The drug degradation outcomes for each condition of the query molecule, as predicted by Zeneth, are summarized in Table 4. The Zeneth program accurately predicted all of the observed DPs.

**Table 4:** Number of degradation products in each stress condition predicted by Zeneth software for Belumosudil.

Condition	Number of degradants predicted by Zeneth
Acidic	57
Basic	86
Oxidative	51
Thermal	66
Photolytic	51

#### 3.6.2 Practical drug degradation assessment

**Fig. 12:**
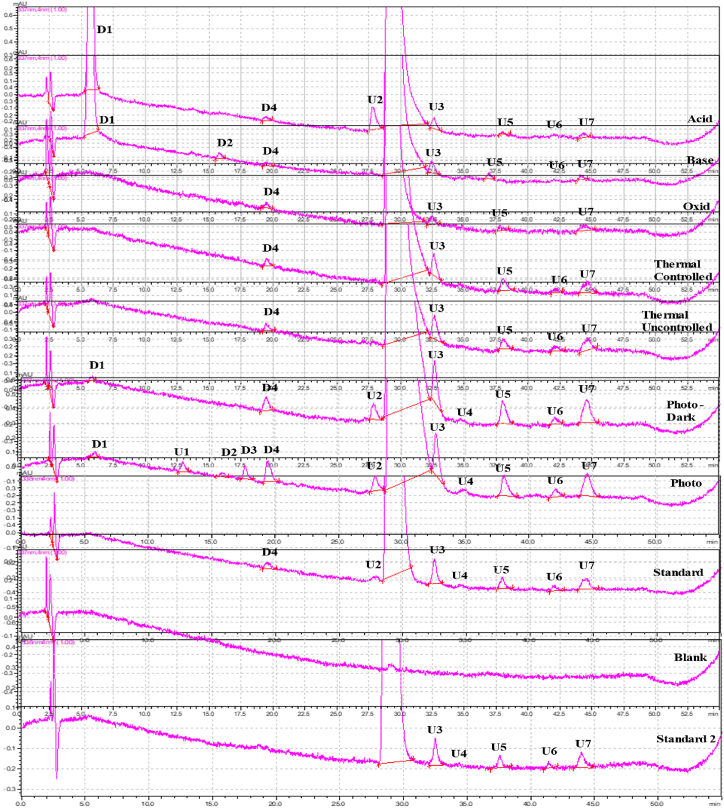
UV chromatogram at a flow rate of 1.1 mL/min and pH of 5.6 of the different stress conditions of the Belumosudil and its degradation products.

All of the stress degradation samples' final chromatograms are shown in Fig. 12. The existence of DPs was confirmed by injecting each sample that was undergoing stress degradation separately. Under different stress conditions, the drug showed four separate DPs, which are D1, D2, D3, and D4. Table 5 provides the percentages of the proportions of the total area specified to each DP.

**Table 5:** The proportions (as percentage) of degradation products under different stress conditions.

Condition	D1	D2	D3	D4
Acidic	5.26 %	–	–	0.072 %
Basic	7.62 %	0.087 %	–	0.064 %
Oxidative	–	–	–	0.12 %
Thermal uncontrolled	–	–	–	0.06 %
Thermal controlled	–	–	–	0.061 %
Photo	0.02 %	0.039 %	0.016 %	0.052 %
Photo control	0.011 %	–	–	0.068 %
Standard	–	–	–	0.03 %

The compounds from U3, U5-U7 are impurities found in the standard and are unrelated to the degradation process. U1, U2 and U4 are degradation products resulting from the presence of contaminants, and they are not associated with BEL.

D1 was mostly seen during both acidic and basic hydrolysis, as well as to a lesser extent during photolytic degradation. It is resulted when acyclic carboxamides undergo hydrolysis to provide carboxylic acids (D1) and amines. The reaction is facilitated by both acid and base in the presence of water. While for D2 formation, pyridine molecules with an alkylamino leaving group in the 2-position may undergo hydrolysis in an aromatic nucleophilic substitution process, resulting in the formation of the corresponding 2-pyridones. The reaction is facilitated by the presence of both acid and base, as well as water. This transformation involves aromatic rings with several nitrogen atoms, including converting pyrimidine into the equivalent pyrimidone. These substrates are particularly favorable for the reaction. In one of the mesomeric forms, the nitrogen and the heterosubstituted carbon atom must be connected by a double bond for the reaction to occur. The scope of the transformation does not include effective leaving groups like halides and esters, since these are addressed by a separate transformation in Zeneth software (version 9). A different transition covers groups such as nitro and sulfonyl, which may also function as leaving groups.

**Fig. 13:**
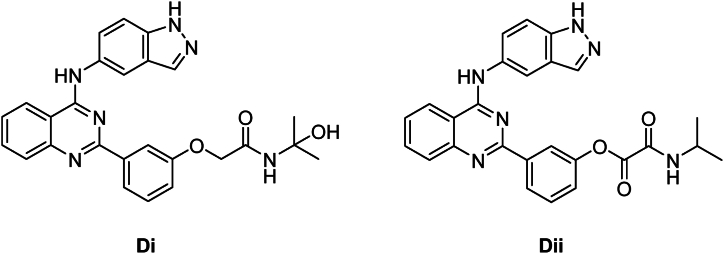
Chemical structure of Di and Dii.

The formation of both D3 and D4 was not directly from the query molecule itself, but rather from intermediate DPs, Fig. 13. The oxidation of BEL N-alkyl amide is initiated by light or molecular oxygen to give hemiamidal (Di). However, Dii formation from query molecule involves oxidation by molecular oxygen (autoxidation). In this transformation, the electron-donating group (amine) and the electron-withdrawing group (carbonyl) initiate a reaction which abstracts the hydrogen atom from the methylene carbon atom to yield a hydroperoxide. This hydroperoxide then undergoes elimination to give an alpha-carbonyl carboxylic acid or derivative as the product.

Acyclic hemiaminals, also known as carbinolamines, undergo rapid fragmentation to produce carbonyl compounds and amines. This process can be observed in the creation of D3 from Di. The process is facilitated by both acidic and basic hydrolysis and may occur spontaneously. The hydrolysis of acyclic carboxylic aryl esters, as seen in D4 formation from Dii, produces carboxylic acids and aromatic alcohols. This reaction may be accelerated by both acid and base. Base catalysis occurs when a hydroxide ion attacks the carbonyl carbon by nucleophilic attack, whereas acid catalysis happens when the carbonyl oxygen is protonated, making the carbonyl carbon more vulnerable to nucleophilic attack by water. D4 was detected in the standard solution at a low concentration when the standard was injected 6 h after preparation, but not detected when injected before (Table 6).

**Table 6:** Practically observed and theoretically predicted degradation products by Zeneth software for Belumosudil.

Name	Parent	Score	Transformation Name	Conditions	Exact Mass
D1	Q	699	Hydrolysis of amide	Water; pH	411.1331
D2	Q	699	Hydrolysis of 2-heterosubstituted pyridine or related compound	Water; pH	337.1426
D3	Di	600	Cleavage of acyclic hemiaminal, hemiacetal or hemimercaptal	None	410.1491
D4	Dii	862	Hydrolysis of aryl ester	Water; pH	353.1277

Q = The parent.

The Zeneth drug degradation prediction showed that the degradation of BEL is mainly caused by the hydrolysis of amide, 2-heterosubstituted pyridine or similar compounds, or aryl ester. Oxidation also plays a secondary role in the formation of Di and Dii by oxidizing alpha-carbonyl amide or ether. The primary factors contributing to Di and Dii instability/invisibility are oxidative processes, followed by hydrolysis.

**Fig. 14:**
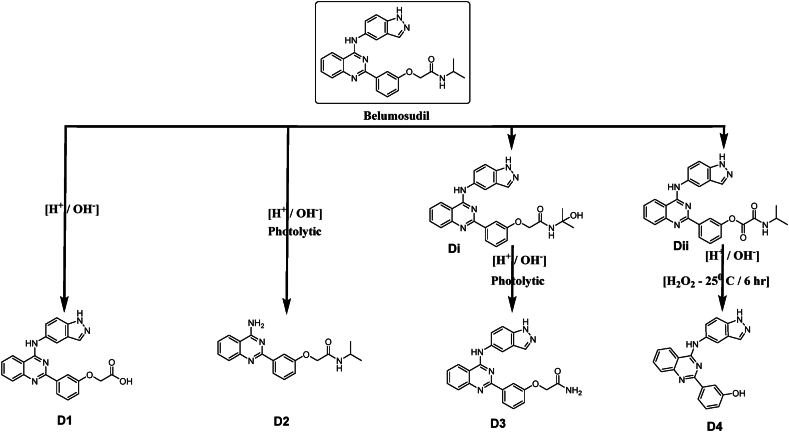
The proposed degradation pathway of Belumosudil under various forced stress conditions.

The proposed degradation pathway of BEL under various forced stress conditions is represented in Fig. 14.

### 3.7 UPLC-TQD-MS/MS for the characterization of DPs

The degradation of BEL was studied systematically under various stress conditions, including hydrolytic, oxidative, thermal, and photolytic stress. During these stress conditions, four DPs were observed. The comprehensive characterization and identification of these DPs, especially when they are not present in pure form, is a significant problem. To address this, a thorough and rigorous strategy was taken, depending on the capabilities of online UPLC-ESI MS/MS. This analytical approach enabled a thorough investigation of the degradation products, revealing their structural characteristics and fragmentation patterns. The findings of this work are shown in Table 7, and they provide important insights into the degradation routes and intermediates of BEL under diverse stressors. This systematic method helps to provide a more comprehensive knowledge of BEL's stability profile, which is critical for guaranteeing the quality and effectiveness of pharmaceutical formulations incorporating BEL.

**Table 7:** Belumosudil and its degradation products MS data, along with the electrospray ionization positive mode product ions and their corresponding proposed degradation pathways.

ID	*m*/*z*	MS/MS Fragment Ions	Proposed Degradation Pathway
BEL	453	366, 353, 325, 221	–
D1	412	338, 325, 296, 192	Hydrolysis
D2	337	222, 209, 193, 94	Hydrolysis
D3	411	325, 295, 234, 193	Oxidation-Hydrolysis
D4	354	325, 284, 221, 192, 93	Oxidation-Hydrolysis

#### 3.7.1 Behavior of protonated BEL in MS/MS fragmentation analysis

**Fig. 15:**
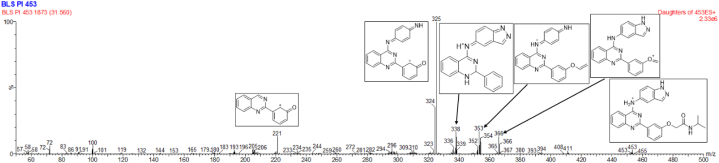
ESI-MS fragmentation spectrum of ([M + H]^+^) ion of Belumosudil (*m/z* 453).

At *m/z* 453, the ESI-TQD spectrum of BEL showed a positive mode peak that corresponds to a protonated molecular ion. C_26_H_25_N_6_O_2_^+^ is the probable chemical formula for this ion. The presence of product ions was revealed by the ESI-MS/MS analysis of the protonated molecular ion ([M + H]^+^) of BEL at *m/z* 366, 353, 338, 325, and 221, Fig. 15. By combining the MS/MS data with the most probable formulas of the product ions, which were obtained from the *m/z* readings, the predicted drug fragmentation pathway was established. Figs. S1–S4 showed the structural representation of DPs' fragment ions (as seen in the supplementary file).

### 3.8 Validation of the developed method

A validation approach was conducted following ICH guideline Q2 (R1) for the newly developed stability indicating method. A PDA detector was used to assess the selectivity of this method by analyzing the peak purity of BEL. The results confirmed that the BEL's purity was sufficient, and the method demonstrated selectivity (Fig. S5) as seen in the supplementary file. The linearity of the concentration was successfully confirmed and validated over the whole concentration range of 5.0–120.0 μg/mL. A linear regression model was used for statistical analysis of the data collected from the calibration curve. The linear regression equation was determined to be y=14569x–58036, with a correlation coefficient of 0.9997.

Measurements were evaluated for precision within one day and throughout three days at concentrations of 8.0, 60.0, and 110.0 μg/mL. Each concentration was tested three times on the same day and consecutive days. The intraday and interday precision studies showed a %RSD values below 2 % (Table 8), demonstrating a high degree of precision in the suggested method. The accuracy of the BEL assay was evaluated by testing a standard sample at five different concentration levels: 8.0, 30.0, 60.0, 90.0, and 110.0 μg/mL. Each level was replicated three times to enhance accuracy and reduce experimental error. The %RSD values ranged from 0.04 % to 0.71 %. The results of the system suitability parameters are shown in Table 9.

**Table 8:** Parameters of regression and validation of the optimized HPLC method for Belumosudil.

Parameter	BEL
Linearity range (μg/mL)	5.0–120.0
Slope	14569.0
Intercept	−58036.0
Correlation coefficient	0.9997
Accuracy (mean ± SD)	101.57 ± 0.43
%RSD	0.42
Precision^a^ (RSD)	1.51
Intermediate precision^b^ (RSD)	1.63
LOD (μg/mL)	1.5
LOQ (μg/mL)	4.95

(a) Intraday precision (average of three replicates at three different concentrations of each (n = 9) within the same day); the concentrations were (8, 60, 110 μg/mL) of BEL.

(b) Interday precision (average of three replicates at three different concentrations of each (n = 9) repeated on three successive days); the concentrations were (8, 60, 110 μg/mL) of BEL.

**Table 9:** Parameters of system suitability for the optimized HPLC method.

Parameters	D1	D2	D3	D4	BEL	Reference Value^a^
Resolution R_s_	10.3	5.3	3.5	2.7	2.1	Rs ≥ 2
Selectivity α	1.4	5.6	6.8	7.7	12	1 < K < 10
Tailing factor	0.91	1.2	1.8	1.3	1.6	T ≤ 2
Column efficiency (N)	4452.2	16131.7	18503.3	8598.6	23089.9	N˃2000
HETP^b^	56.15	3.87	13.51	29.07	10.83	

a USP reference.

b Height equivalent to theoretical plate (μm).

To determine the optimized method's reliability in the face of deliberate changes to the methodology's parameters, the method's robustness was evaluated. The QbD approach and the Design Expert software (version 12) were used to effectively identify a robust zone without doing extra experiments outside that zone. By selecting three confirmation points inside the design space, we were able to evaluate the predicted robust region's robustness. Even when the procedure settings were changed, the results of all responses were found to be unaltered. According to Table 10, this outcome indicates that the method showed robustness within the given design area.

**Table 10:** Robustness results of the optimized HPLC method for Belumosudil and its degradation products.

Confirmation Location #1								
Slope	FR	pH						
55	1.0	5.5	Statistical Parameters					
Response	Mean	Median	SD	n^a^	SE	95 % low	Mean	95 % high
Resolution of API and PI	4.84	4.84	0.13	4.00	0.09	4.65	4.73	5.03
Rt of LEP	47.35	47.35	0.34	4.00	0.26	46.80	47.54	47.90
K factor for 1st Peak	1.39	1.39	0.03	4.00	0.03	1.34	1.44	1.45
**Confirmation Location #2**								
Slope	FR	pH						
57	1.1	5.6	Statistical Parameters					
Response	Mean	Median	SD	n^a^	SE	95 % low	Mean	95 % high
Resolution of API and PI	4.80	4.80	0.13	4.00	0.08	4.62	4.66	4.97
Rt of LEP	44.13	44.13	0.34	4.00	0.22	43.66	44.48	44.60
K factor for 1st Peak	1.37	1.37	0.03	4.00	0.02	1.33	1.35	1.42
**Confirmation Location #3**								
Slope	FR	pH						
60	1.2	5.7	Statistical Parameters					
Response	Mean	Median	SD	N^a^	SE	95 % low	Mean	95 % high
Resolution of API and PI	4.70	4.70	0.13	4.00	0.09	4.65	4.73	5.03
Rt of LEP	40.54	40.54	0.34	4.00	0.26	46.80	47.54	47.90
K factor for 1st Peak	1.29	1.29	0.03	4.00	0.03	1.34	1.44	1.45

a Average of four determinations.

### 3.9 In silico prediction of toxicity and mutagenicity of BEL and its DPs

The DEREK (version 6.2.1) software was used to assess the mutagenicity and toxicity of BEL and its DPs. Table 11 displays the results of the mutagenicity and toxicity tests conducted on them. Predictions were based on a wide variety of parameters, including but not limited to: people, guinea hamsters, dogs, rabbits, primates, rats, microbes, and Salmonella typhimurium. Several endpoints were calculated, including carcinogenicity, phospholipidosis, skin sensitization, nephrotoxicity, ocular toxicity, hepatotoxicity, mutagenicity, neurotoxicity, phototoxicity, and other endpoints. The purpose of DEREK's study was to evaluate the BEL against the toxicity projections made by each DP. Presented below are the results: D1 was anticipated to produce hepatotoxicity. The presence of phenoxyacetic acid or its derivatives causes this compound's activity. D4 was predicted to produce skin irritation and, to a lesser degree in terms of probability, skin sensitization as a result of the presence of phenol or substituted phenol. No toxicity warnings were projected in BEL, D2, or D3.

**Table 11:** Prediction of toxicity and mutagenicity of Belumosudil and its degradation products.

Drug/DP No.	Structural Alert Code	Structural Alert	Endpoints for Toxicity
Drug	No alerts were found for toxicity		
D1	690		Hepatotoxicity
D2	No alerts were found for toxicity		
D3	No alerts were found for toxicity		
D4	915		Skin irritation
	439		Skin sensitization

## 4 Conclusions

An HPLC technique utilizing reversed-phase chromatography was developed to quantify BEL, employing a Quality by Design (QbD) approach. Before the research process began, there were no known stability-indicating analytical techniques for BEL, and no BEL DPs were available. Therefore, the use of forced degradation samples has become a significant technique in the QbD framework. A mathematical approach was created to examine the CQAs linked to ATP. A method was suggested for creating a flexible area inside the control space of the design region. Using a mathematical model helps to provide a thorough comparison of how process factors affect results. The analytical method was validated at the selected operating point inside the control space to evaluate its accuracy, repeatability, sensitivity, and linearity. The developed method successfully met the specified acceptance criteria for ATP set at the beginning of the QbD process. The Zeneth Nexus® program was initially executed to predict DPs under various stress situations. The established LC technique efficiently separates four unique DPs: D1, D2, D3, and D4. In addition, the approach was extended to include LC-MS, which helped in analyzing and determining the structural features of various DPs. The results were compared to that from Zeneth software to establish the possible degradation pathway. Furthermore, the in silico toxicity of BEL and its DPs was assessed using DEREK software. Various adverse outcomes, such as hepatotoxicity and skin sensitization, were estimated with probability in two DPs (D1 and D4). Acquiring the DPs is possibly the next task at hand. The suggested technique may be used in quality control labs to quantitatively determine and act as the SIAM for BEL.

## Ethics statement

Not applicable.

## Data availability statement

All data are available within the manuscript and the supplementary file.

## CRediT authorship contribution statement

**Awadh M. Ali:** Writing – original draft, Supervision, Resources, Methodology, Funding acquisition, Data curation. **Mohammed M. Alanazi:** Writing – review & editing, Visualization, Supervision, Software, Project administration, Investigation, Formal analysis. **Mohamed W. Attwa:** Writing – review & editing, Validation, Software, Investigation, Formal analysis. **Ibrahim A. Darwish:** Writing – review & editing, Validation, Supervision, Methodology, Formal analysis, Data curation. **Hany W. Darwish:** Writing – review & editing, Visualization, Supervision, Software, Project administration, Investigation, Formal analysis, Conceptualization.

## Declaration of competing interest

The authors declare that they have no known competing financial interests or personal relationships that could have appeared to influence the work reported in this paper.

## Abbreviations

BEL: Belumosudil
GVHD: graft-versus-host disease
QbD: Quality by Design
CQAs: Critical Quality Attributes
CPPs: Critical Process Parameters
UV: Ultraviolet
MS: Mass Spectrometry
DoE: Design of Experiments
HPLC: high-performance liquid chromatography
GC: gas chromatography
DPs: degradation products
APIs: active pharmaceutical ingredients
PDA: Photodiode Array detector
ATP: Analytical Target Profile
DS: Design Space
OFAT: One Factor at a Time
FDS: fraction of design space
