# Supplementary material for: Comprehensive chromatographic analysis of Belumosudil and its degradation products: Development, validation, and In silico toxicity assessment
Source: Heliyon. 2024 Sep 24;10(19):e38369. doi: 10.1016/j.heliyon.2024.e38369 (PMC11466582; doi:10.1016/j.heliyon.2024.e38369)
Supplement: Multimedia component 1 [file mmc1.docx]

**Supplementary Information**

**Comprehensive Chromatographic Analysis of Belumosudil and its Degradation Products: Development, Validation, and In Silico Toxicity Assessment**

Awadh M. Ali*, Mohammed M. Alanazi, Mohamed W. Attwa, Ibrahim A. Darwish and Hany W. Darwish

Department of Pharmaceutical Chemistry, College of Pharmacy, King Saud University, P.O. Box 2457, Riyadh 11451, Saudi Arabia; aali1@ksu.edu.sa (A.M.A.); mmalanazi@ksu.edu.sa (M.M.A.); mzeidan@ksu.edu.sa (M.W.A); idarwish@ksu.edu.sa (I.A.D.); hdarwish@ksu.edu.sa (H.W.D.)

*Correspondence: aali1@ksu.edu.sa; Tel.: +966- 1146-77343; Fax: +966-1146-76220

**S.1. In Silico Degradation Prediction**

Zeneth Nexus^®^ (version 9.2.1) by Lhasa is an in silico software used to predict degradation pathways of drug molecules based on their chemical structure. While it may overestimate DPs compared to real experiments, it is a key tool in drug development for identifying potential degradation issues. The program analyzes chemical structures, reaction conditions, and other parameters to generate a degradation profile under various conditions (e.g., hydrolysis, oxidative, thermal, photolytic). This profile serves as a guideline for practical experiments, outlining potential DPs with associated likelihood scores. Zeneth Nexus^®^ also integrates *m/z* data from LC-MS to predict primary and secondary DPs. The software employs a reasoning search and a continuously updated knowledge library to assess degradation probabilities, with scores ranging from 0 (impossible) to 1000 (certain). The latest version offers a continuous scale for more refined predictions, enhancing its utility in evaluating degradation risks during drug production. Zeneth Nexus^®^ has the following limitations:

1- When the number of degradation products exceeds the user-defined maximum, some degradation products may exceed the cutoff limit and be excluded from the prediction results.

2- Incomplete predictivity arises with Zeneth when the query compound belongs to a different structural class than the one on which the transformation scope is initially based, leading to limited predictive accuracy.

3- Excessive overprediction can undermine both the accuracy and clarity of the prediction results.

4- Maximum number of DPs produced is 1000.

**S.2. In Silico Toxicity Prediction**

DEREK Nexus^®^ is a software tool developed by Lhasa to assess the mutagenicity and carcinogenicity of small organic molecules. DEREK Nexus^®^ relies on knowledge databases to predict various toxicities (e.g., mutagenicity, carcinogenicity, teratogenicity). This in silico tool aid in the qualitative structure-activity relationship (QSAR) analysis, and its outputs can inform further research and integration into ICH M7 guidelines. Predictions are initiated by inputting the chemical structure of the query molecule and its DPs.

# S.3. MS/MS fragmentation pattern of DPs of Belu

The MS spectra of the primary DP (D1) reveal an elemental formula of C_23_H_18_N_5_O_3_^+^ with the ([M + H]^+^) ion peak at *m/z* 412. Figure 1S depicts the fragmentation process that gave support for the suggested structure. D1's protonated molecular ion ([M + H]^+^) produced distinct product ions in its fragmentation spectra at *m/z* 338, 325, 296, and 192. These identified ions played a crucial role in determining the potential structure for D1.


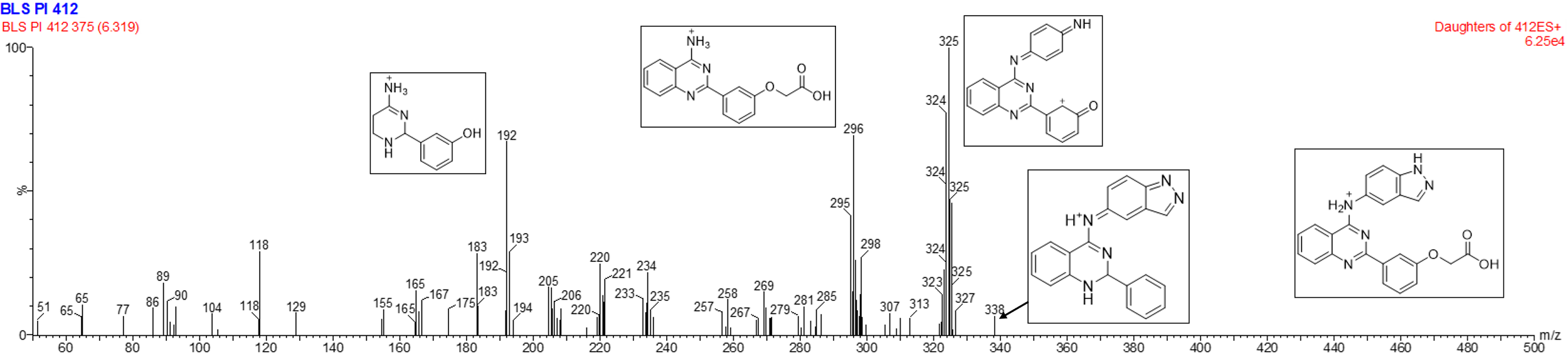


**Fig. 1S.** ESI-MS fragmentation spectrum of ([M+H]^+^) ion of D1 (*m/z* 412).

Figure 2S shows the fragmentation spectrum of compound C_19_H_21_N_4_O_2_^+^ (D2) with product ions having *m/z* values of 222, 209, 192, and 94. The ions had a crucial role in deciding the most likely suggested structure.


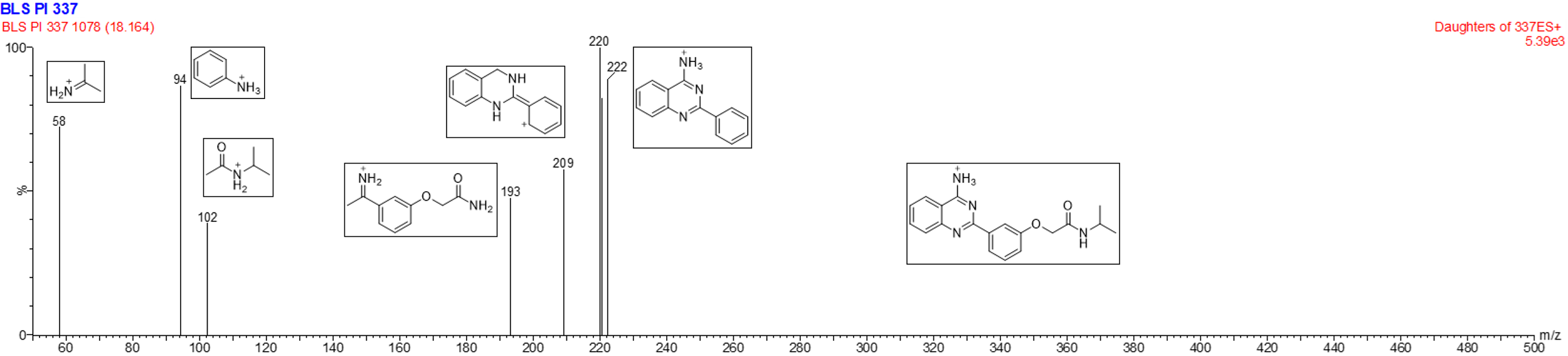


**Fig. 2S.** ESI-MS fragmentation spectrum of ([M+H]^+^) ion of D2 (*m/z* 337).

Figure 3S shows the mass fragmentation spectrum of the ([M + H]^+^) ion (*m/z* 411) of D3 and its elemental formula (C_23_H_19_N_6_O_2_^+^), revealing product ions at *m/z* 325, 295, 234, and 193. The data suggest that D3 is equivalent to 2-(3-(4-((1H-indazol-5-yl)amino)quinazolin-2-yl)phenoxy)acetamide.


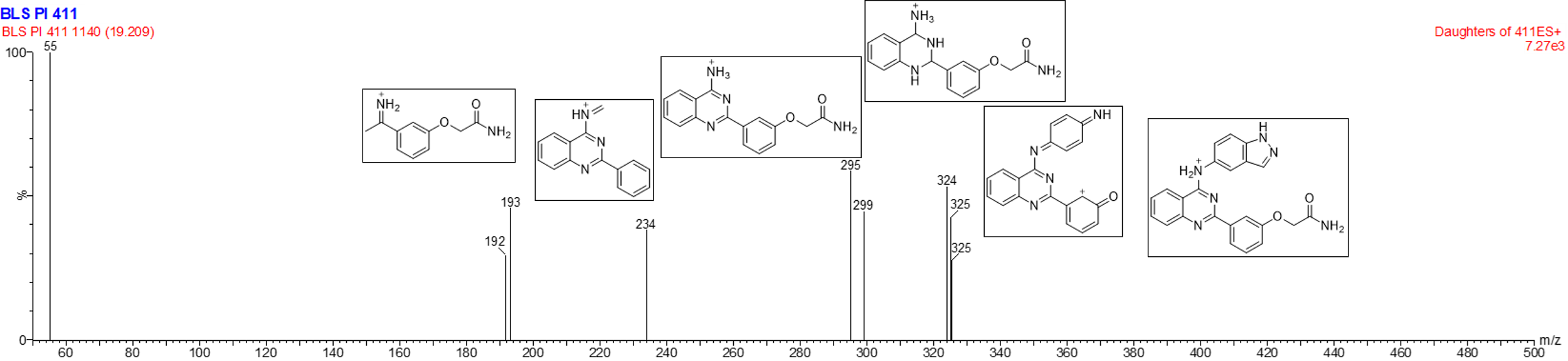


**Fig. 3S.** ESI-MS fragmentation spectrum of ([M+H]^+^) ion of D3 (*m/z* 411).

The molecular structure of D4 was determined based on *m/z* observations of the MS/MS product ion spectrum. The suggested structure is supported by the presence of product ions at *m/z* 325, 284, 221, 192 and 93, Figure 4S. The suggested most likely configuration of D4 is 3-(4-((1H-indazol-5-yl)amino)quinazolin-2-yl)phenol.


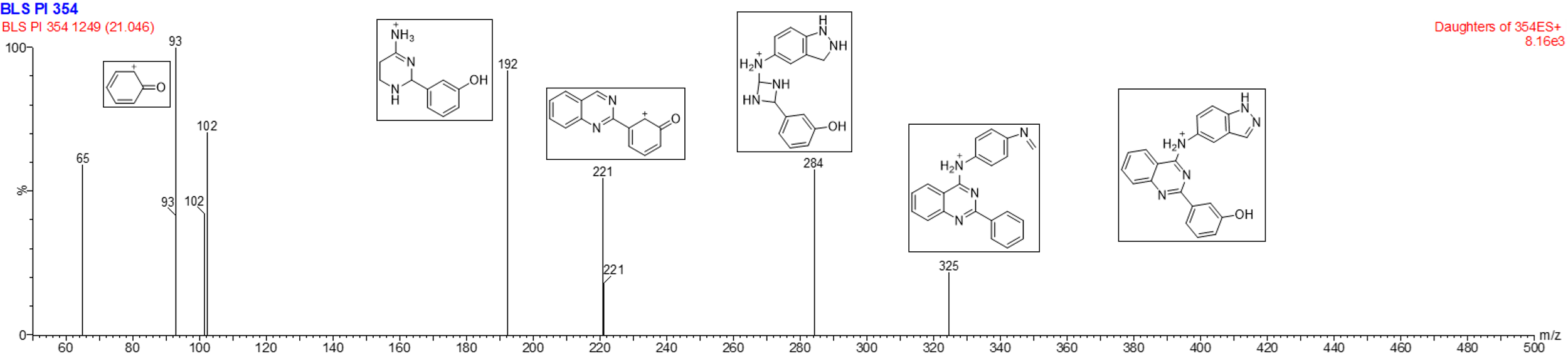


**Fig. 4S.** ESI-MS fragmentation spectrum of ([M+H]^+^) ion of D4 (*m/z* 354).


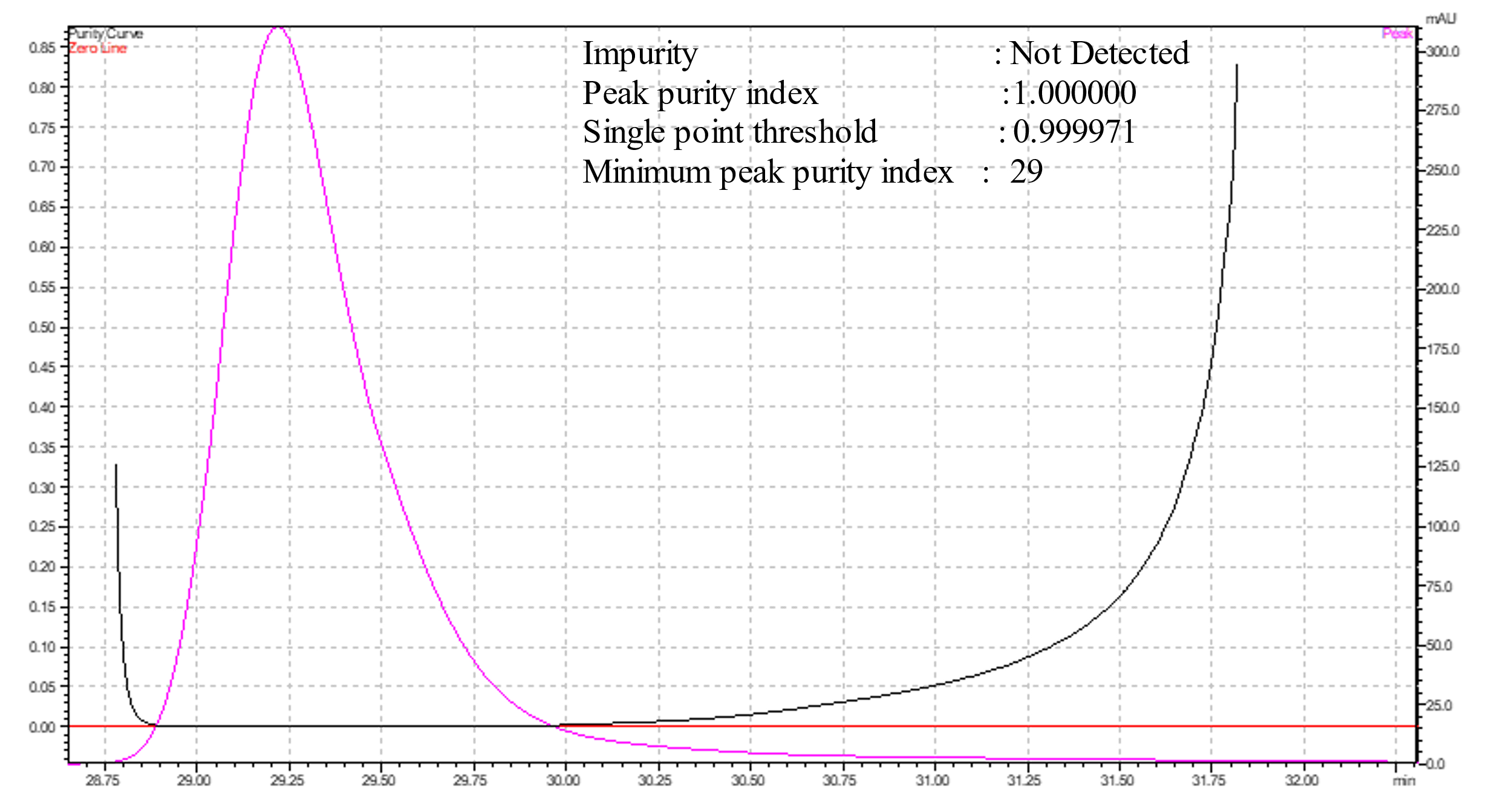


**Fig. S5**: Purity plot of Belumosudil.
